# Targeting interferon signaling and CTLA-4 enhance the therapeutic efficacy of anti-PD-1 immunotherapy in preclinical model of HPV^+^ oral cancer

**DOI:** 10.1186/s40425-019-0728-4

**Published:** 2019-09-18

**Authors:** Stephanie Dorta-Estremera, Venkatesh L. Hegde, Ravaen B. Slay, Rachel Sun, Ananta V. Yanamandra, Courtney Nicholas, Sita Nookala, Gloria Sierra, Michael A. Curran, K. Jagannadha Sastry

**Affiliations:** 1 Department of Immunology, The University of Texas MD Anderson Cancer Center, Houston, TX 77030 USA; 2 The University of Texas MD Anderson Cancer Center UTHealth Graduate School of Biomedical Sciences at Houston, Houston, TX 77030 USA; 3 Oncology Research for Biologics and Immunotherapy Translation, The University of Texas MD Anderson Cancer Center, Houston, TX 77030 USA

**Keywords:** Abscopal, Checkpoint blockade, CD8^+^ T cells, HNSCC, HPV, Immunotherapy, MDSC, STING

## Abstract

**Background:**

The US is experiencing an epidemic of HPV^+^ oropharyngeal cancers (OPC), the rates and burden of which now exceed that for cervical cancer. Immunotherapy targeting programmed death 1 (PD-1) on tumor-infiltrating lymphocytes and/or its ligand PD-L1 on tumor cells, which was effective in several cancers has however, showed efficacy in only less than 15% of patients.

**Methods:**

We used a preclinical HPV^+^ oral tumor model, mEER, consisting of mouse tonsil derived epithelial cells expressing HPV-16 E6 and E7 genes, along with the H-ras oncogene to test strategies for enhancing the efficacy of anti-PD-1 therapy.

**Results:**

Monotherapy with PD-1 blocking antibody was ineffective against flank-implanted tumors, but induced regression in 54% of mice bearing orthotopic tongue tumors that correlated with higher CD8 T cell responses. Since the CD8^+^ T cells derived from tongue tumors also showed high levels of the immune checkpoint inhibitory receptor CTLA-4, we tested combination immunotherapy targeting both CTLA-4 and PD-1 together and observed 93.3% survival of mice bearing tumors in the tongue for the duration of our 100-day study. Protective immunity correlated with a significant decrease in immunosuppressive lymphoid and myeloid populations within the tumor microenvironment. Consistent with the reported capacity of interferon-driven PD-L1/PD-1 pathway induction to serve as a biomarker of response to PD-1 blockade, we observed elevated interferon signaling and significantly higher levels of PD-1/PD-L1 in tongue-implanted mEER tumors compared to those growing on the flank correlating with their preferential responsiveness to PD-1 blockade. More importantly, in a pseudometastasic mouse model bearing both flank and tongue tumors to represent metastatic disease, delivery of Stimulator of Interferon Induced Genes (STING) agonist into the flank tumors combined with systemic treatment with α-PD-1 and α-CTLA-4 antibodies resulted in sustained tumor regression in 71% of mice. In this case, productive abscopal anti-tumor immunity was associated with robust increases in the ratios of cytotoxic CD8^+^ T cells (CTL) versus regulatory T cells (Treg) and versus functional myeloid-derived suppressor cells (MDSC).

**Conclusions:**

These results support combining α-PD-1 therapy with induction of IFN-α/β signaling via provision of STING agonist and/or through CTLA-4 blockade as potential treatment option for HNSCC patients, especially, those not responding to α-PD-1 monotherapy.

## Background

The incidence of oropharyngeal cancers, a subset of head and neck squamous cell carcinomas (HNSCC), associated with Human papillomavirus (HPV) has been significantly on the rise in the United States and other developed countries [1]. The HPV^+^ HNSCC patients are younger with tumors typically in the tonsillar region or base of tongue, lymph node involvement and with higher risk of advanced metastatic disease when compared to HPV^−^ patients [2]. The HPV^+^ tumors are also pathologically distinct with increased lymphocyte infiltration within the tumor microenvironment. While immune checkpoint therapy (ICT) is effective in HPV^+^ patients [1, 2], the response rate is still low (< 15%) and achieving the curative efficacy in majority of the patients remains an unmet medical need [3]. Therefore, strategies targeting multiple immune checkpoints alone or in combination with therapeutic vaccines and/or targeted therapies are of critical interest in this area.

Upon activation, tumor-infiltrating T cells express immune checkpoint receptors such as programmed death-1 (PD-1) to maintain self-tolerance. In the tumor microenvironment, high expression of PD-1 on CD8^+^ T cells indicates engagement of an “off switch” suggesting deficient effector function and poor expansion and memory potential. Furthermore, sustained PD-1 expression is often associated with expression of multiple inhibitory receptors leaving T cells dysfunctional within the immunosuppressive tumor microenvironment [4–6]. In this setting, blockade of PD-1 has evolved into the treatment of choice to preserve and restore the function of anti-tumor T cells. For multiple cancers, α-PD-1 immunotherapy has proven successful in enhancing effector CD8^+^ T cell responses and overcoming the immunosuppressive tumor microenvironment. In case of head and neck squamous cell carcinoma (HNSCC), expression of PD-1 ligands, PD-L1 and PD-L2, within the tumor has been correlated with poor prognosis, suggesting that α-PD-1 therapy is a relevant approach to anti-tumor immunity [7–9]. However, because of the low response rate to anti-PD1 therapy in HNSCC patients [3], it is important to better understand the tumor microenvironment of HNSCC in order to elucidate precise mechanisms of resistance to α-PD-1 therapy, and to design supplemental treatments or combination strategies to reverse α-PD-1 non-responsiveness and improve clinical outcome.

Previously, we reported that immunotherapy using α-4-1BB, α-CD40, or α-CTLA-4 showed modest single-agent efficacy against HPV-16 E6/E7^+^ tonsillar-epithelia derived mEER tumors implanted subcutaneously on the flank of syngeneic C57BL/6 J mice [10]. While various combinations of α-4-1BB, α-CD40 or α-CTLA-4 antibodies were significantly more efficacious compared to single-antibody treatments, therapeutic efficacy was enhanced when immunotherapy treatments were combined with intranasal E6/E7 peptide vaccine [11]. However, PD-1 blockade, demonstrated little to no efficacy against subcutaneous mEER tumors [11]. In contrast to flank-implanted mEER tumors, we observed that α-PD-1 and α-CTLA-4 demonstrated the greatest single-agent efficacy in treating these HPV^+^ oropharyngeal tumors implanted in the tongue. Since the oral cavity contains a distinct immune infiltrate relative to other systemic tissues [12, 13], we sought first to identify the cell populations responsible for differential α-PD-1 therapy responsiveness, and second to investigate effective strategies to improve responses to α-PD-1.

## Methods

### Animals

Male C57BL/6 mice (5–10 weeks) were purchased from the Jackson Laboratories and were maintained in a pathogen-free environment. Animal studies were pre-approved and carried out in accordance with University of Texas MD Anderson Cancer Center Institutional Animal Care and Use Committee (IACUC) guidelines. Animals were anesthetized with isoflurane for tumor inoculations and blood draws, and euthanized according to IACUC guidelines.

### Cell line

Mouse tonsil epithelial cells expressing HPV-16 E6 and E7 and H-Ras (mEER) were a kind gift from Dr. J. Lee (NantKwest Inc., Culver City, CA). These cells were maintained in complete media as previously described [14], and sub-cultured at 80% confluence the day before tumor induction in mice.

### Reagents

Tumor-infiltrating lymphocytes (TIL) were analyzed by 16-color multi-parametric flow cytometry using the following antibodies: BUV737 anti-CD3 (17A2), Alexa Fluor 700 anti-Granzyme B (GB11), BV605 anti-CD11c (HL3), APC-Cy7 anti-CD11b (M1/70), anti-mouse CD16/32 (2.4G2, mouse Fc-block) from BD Bioscience (San Jose, CA), BV650 anti-CD8 (53–6.7), APC anti-CTLA-4 (UC10-4B9), PerCP-ef710 anti-Lag3 (C9B7W), PE-Cy7 anti-PD-1 (RMP1–30), BV711 anti-PD-L1 (10F.9G2), PE-Cy5 anti-F4/80 (BM8) from Biolegend (San Diego, CA), Alexa Fluor 488 anti-FoxP3 (150D/E4) and e450 anti-Gr-1 (RB6-8C5) from eBioscience (Waltham, MA). The following antibodies for in vivo administration were purchased from BioXcell (West Lebanon, NH) and used at the concentrations shown: α-PD-1 (RMP1–14 at 250 μg per dose), α-CTLA-4 (9H10 at 100 μg per dose) and α-Lag-3 (C9B7W at 200 μg per dose). The STING agonist ML-RR-S2 CDA (ADU-S100) was procured from MedChemExpress (Monmouth Junction, NJ). For fluorescence immunohistochemistry, rabbit monoclonal anti-mouse PD-L1 antibody was purchased from Abcam (Cambridge, MA) and chicken anti-rabbit IgG cross-absorbed antibody Alexa Fluor 594 conjugate from Invitrogen (Carlsbad, CA).

### In vivo tumor challenge

Mice were implanted with 4 × 10^4^ mEER cells in 50 μl PBS into the base of the tongue or 1 × 10^6^ mEER cells in 200 μl PBS subcutaneously in the flank. Mice were monitored closely and euthanized when a necrotic tumor was observed and/or when the mice lost 20% or more of their initial weight. For characterization of TILs, mEER cells were mixed in a 2:1 ratio with Matrigel (BD Biosciences, San Jose, CA) and 1 × 10^5^ cells in 50 μl per animal were implanted in the tongue. For the mEER pseudometastasic setting, 4 × 10^4^ cells for survival experiments or 1 × 10^5^ cells for TIL analysis were implanted in the tongue, and 1 × 10^6^ cells subcutaneously in the flank of each mouse.

### Immunotherapy

Starting between days 5 and 7 after tumor challenge mice received intraperitoneal injections of immune checkpoint therapeutic antibodies or their combination, three times at three-day intervals. Control animals were untreated. For the pseudometastasic model, STING agonist (ML-RR-CDA) was given by intratumoral (i.t.) injection at day 10 when tumors had reached at least 4 mm in diameter, and a second time on day 16. The immune checkpoint antibodies were administered i.p. at days 10, 13, 16 for TIL analysis and an additional dose on day 19 for survival experiments.

### Flow cytometry

For characterization of TIL, mice were euthanized at the days specified in the results section. Tongue and flank tumors were collected and digested as previously described [10]. Purified leukocytes were stained for multi-parametric flow cytometry analysis with a 16-color antibody panel. Cells were blocked with mouse Fc-block, stained with surface markers, fixed and permeabilized with the FoxP3 Fix/Perm Kit (eBioscience, Waltham, MA) followed by staining for intracellular markers. Samples were run in an LSR-II X-20 Fortessa (BD Biosciences, San Jose, CA) at the South Campus Flow Cytometry Core, MD Anderson Cancer Center (Houston, TX) and analyzed using FlowJo version 10 (Flowjo LLC, Ashland, OR). The live/dead fixable aqua dye (Thermo Scientific, Waltham, MA) was used to gate out dead cells and to include only live cells for analysis. Live leukocyte gate was set based on forward and side scatter to include both lymphocytes and larger myeloid cells. Tregs were identified based on CD4^+^Foxp3^+^ expression within the CD3^+^ gate. From CD3^−^ gate, CD11b^+^Gr-1^+^ cells were identified as total MDSC population. The ratio of CD8^+^ T cells to Tregs or MDSC were calculated by dividing the percentage of CD8^+^ T cells with that of CD4^+^Foxp3^+^ or CD11b^+^Gr-1^+^ cells.

### Fluorescence immunohistochemistry (IHC)

Freshly harvested tumors were flash frozen in Shandon Cryomatrix embedding resin (Thermo Scientific, Waltham, MA). Cryostat sections (5 μM) were cut and placed on glass slides. The sections were fixed using cold methanol at − 20 °C for 20 min. Blocking of non-specific sites were performed using PBS-based Super block (Thermo Scientific) containing 0.3% Tween-20 for 30 min. The sections were then incubated successively with pre-titrated dilutions of anti-mouse PD-L1 primary antibody (1:250) overnight at 4 °C and chicken anti-rabbit IgG Alexa Fluor 594 conjugate (1:2000) for 1 h at room temperature. The slides were washed between steps using PBS containing 0.1% Tween-20. Sections were mounted using ProLong™ Gold Antifade containing DAPI nuclear counterstain (Molecular Probes, Eugene, OR). Adjacent sections stained with secondary antibody alone were used as staining controls to assess non-specific background. Stained slides were imaged with a fluorescence microscope equipped with digital camera (Olympus USA, Center Valley, PA), and using TRITC (for Alexa Fluor 594) and DAPI filters. Fluorescence photomicrographs obtained from four random regions for each section were analyzed for mean fluorescence intensity for PD-L1 expression using NIH ImageJ software.

### Total RNA extraction

Total RNA was extracted from tumor tissue using PureLink RNA mini kit (Thermo Scientific) according to the manufacturer’s instructions. The quality and concentration of RNA was determined using a NanoDrop UV spectrophotometer, and the RNA integrity was verified using Agilent 2100 BioAnalyzer (Palo Alto, CA).

### RNA-Seq analysis

RNA sequencing was performed using lllumina HiSeq 2000 at the Sequencing and Microarray Facility, MD Anderson Cancer Center (Houston, TX). Fastq files were filtered for Phred quality score of 20 and adapter sequences with minimum length of 35 to remove low quality reads using BBduk BBMap (The U.S. Department of Energy Joint Genome Institute, Lawrence Livermore National Laboratory, Walnut Creek, CA). The mRNA-Seq paired-end reads were aligned onto the mouse genome build UCSC mm10 (NCBI 38) and transcript level quantification of counts was performed using Salmon algorithm [15], followed by differential expression analysis based on a negative binomial distribution model using DESeq2 [16].

### Real-time qPCR

Total RNA were reverse transcribed to obtain cDNA using the iScript cDNA synthesis kit (Bio-Rad, Hercules, CA). Real-time qPCR was performed using target-specific forward and reverse primers and iQ SYBR Green qPCR Supermix using CFX384 Touch real-time PCR detection system (Bio-Rad). Relative quantification was calculated by 2^(−ΔΔCq)^ method and expressed relative to endogenous control 18S. The following mouse primers pairs were used, PD-L1 (CD274): TGC GGA CTA CAA GCG AAT CAC G (forward), CTC AGC TTC TGG ATA ACC CTC G (reverse); Ciita: ACC TTC GTC AGA CTG GCG TTG A (forward), GCC ATT GTA TCA CTC AAG GAG GC (reverse); Mx1: TTC AAG GAT CAC TCA TAC TTC AGC (forward), GGG AGG TGA GCT CCT CAG T (reverse); Isg15: ACG GTC TTA CCC TTT CCA GTC (forward), CCC CTT TCG TTC CTC ACC AG (reverse); Ifng: AAC TGG CAA AAG GAT GGT (forward), GAC CTC AAA CTT GGC AAT AC (reverse); 18S: CCA TTC GAA CGT CTG CCC TAT (forward), GTC ACC CGT GGT CAC CAT G (reverse).

### Liver function assessment

Blood was collected from anesthetized mice through the retro-orbital plexus at day 15 or day 21 after tumor challenge and analyzed for liver enzymes (AST and ALT) at the Clinical Pathology Laboratory in the Veterinary Medicine and Surgery Department at MD Anderson Cancer Center (Houston, TX).

### Magnetic resonance imaging (MRI)

Mice were imaged at day 19 or day 23 after tumor challenge on the 1 T Bruker ICON at the MD Anderson Cancer Center Small Animal Imaging Facility as previously described [10]. Tumor volume was determined in three dimensions with ImageJ software after defining region of interest of the tumor on all possible sections.

### Statistical analysis

All statistics were calculated using GraphPad Prism version 6. Statistical significance was determined using one-way or two-way ANOVA along with post-hoc correction to test differences between multiple groups or Student’s *t*-test to compare two groups. Mantel-Cox log rank test was used to compare survival curves. *P* values less than 0.05 were considered significant.

## Results

### Tumors implanted in the tongue, but not on the flank are sensitive to α-PD-1 therapy

**Fig. 1:**
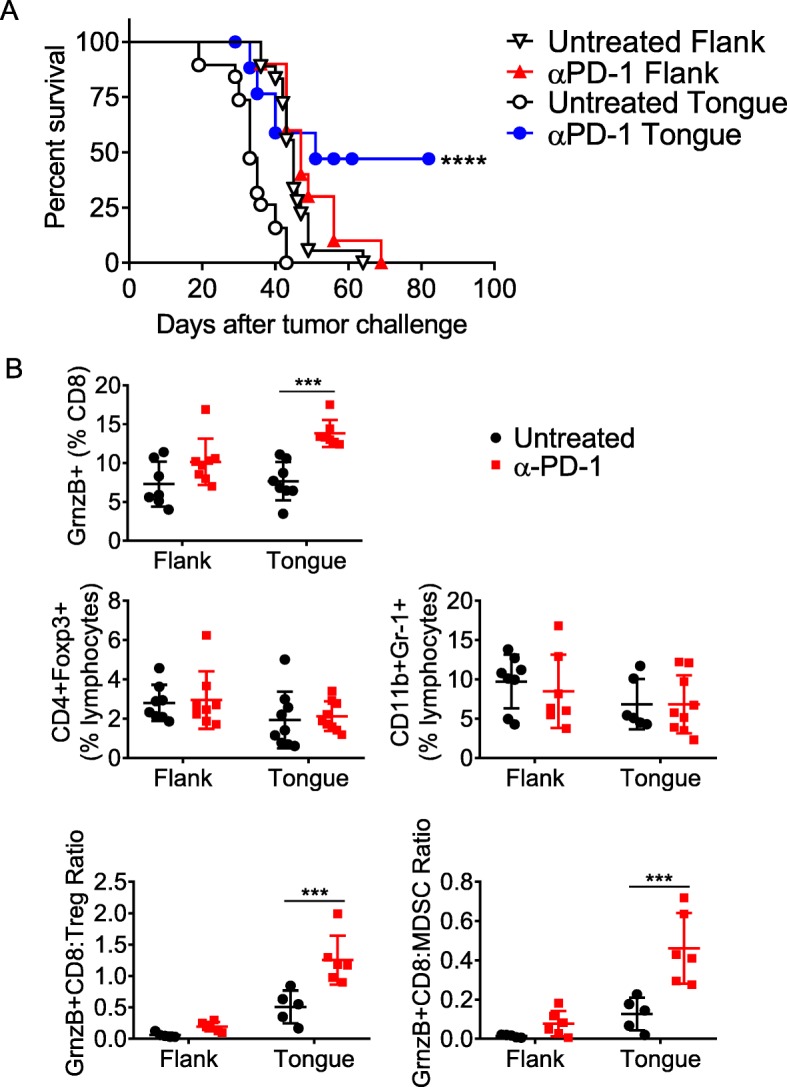
Differential α-PD1 responsiveness of mEER tumors implanted in the flank and tongue. Separate groups of mice were injected with mEER tumor cells in the tongue (4 × 10^4^) or in the flank (1 × 10^6^), and treated with α-PD1 antibodies at days 5, 8 and 11. The percent survival of mice in the different groups is shown (**a**). Mantel Cox-test was performed to determine the significance of survival for each of the treatment groups relative to respective untreated group ****p < 0.00005. Results represent pooled data from multiple experiments (*n* = 10–18 mice/group). **b** At day 15 after tumor implantation mice in the different groups were sacrificed and the TIL were analyzed by flow cytometry to determine the frequencies of Granzyme B expressing functional CD8^+^ T cell populations, CD4^+^Foxp3^+^ Tregs, CD11b^+^Gr-1^+^ MDSC as well as ratios of functional Granzyme B expressing CD8^+^ T cells to Treg and to MDSC

We compared anti-PD-1 responsiveness of mice bearing mEER tumors on the flank to those in the tongue. Tumor bearing mice were treated on days 5, 8 and 11 with α-PD-1 antibody and their survival was monitored. Consistent with our earlier report [11], none of the mice with flank-implanted tumors responded to α-PD-1 therapy while 54% of mice with tongue-implanted tumors exhibited sustained tumor regression with a significant survival advantage (Fig. 1a). The immune correlates for the protective efficacy of α-PD-1 therapy in the tongue tumors included a higher frequency of CD8^+^ T cells, specifically those with cytotoxic potential as evidenced by expression of Granzyme B (CTL). These enhanced T cell frequencies combined with overall pro-inflammatory modulation of the tumor microenvironment also gave rise to elevated ratios of CTL relative to both Tregs and MDSC (Fig. 1b).

**Fig. 2:**
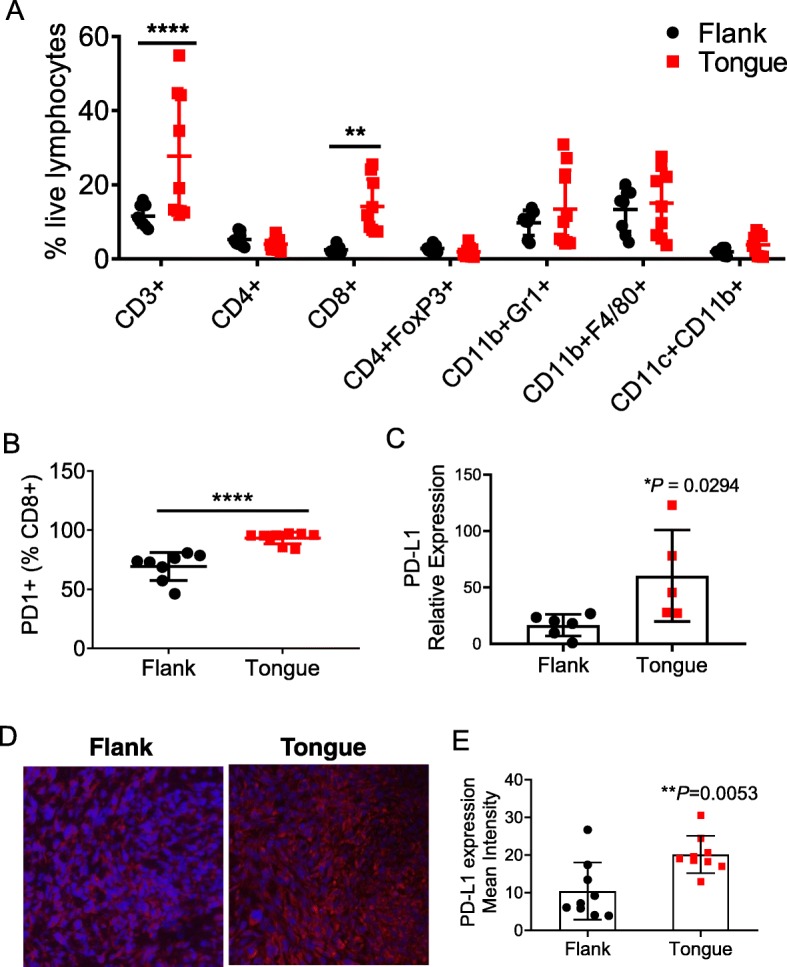
Differential infiltration of T cells between oral and subcutaneous mEER tumors. Tumor-infiltrating leukocytes were isolated at day 15 after tumor implantation from mice bearing flank or tongue mEER tumors and analyzed by flow cytometry. Figure shows percentage of different leukocyte subsets among total live lymphocytes (**a**) and frequencies of PD-1 expressing CD8^+^ T cells (**b**). Results represent pooled data from two separate experiments (*n* = 8–10). Statistical significance was calculated using Two-way ANOVA **p < 0.005, ****p < 0.00005. Flank and Tongue implanted mEER tumors (*n* = 3–6) were analyzed for PD-L1 expression by real-time qPCR (**c**) and fluorescence immunohistochemistry (**d**, **e**). Representative IHC images (**d**) and quantification of PD-L1^+^ cells (**e**) are shown. **p = 0.0053, Student’s t-test

To understand the potential mechanisms for the observed differential α-PD-1 responsiveness of mEER tumors implanted in the flank vs tongue, we first conducted comparative analyses of TIL from the two sites in untreated mice. We observed a significantly higher percentage of T cells (CD3^+^), specifically CD8^+^ T cells, in tongue tumors compared to those on the flank (Fig. 2a). Importantly, a significantly higher frequency of CD8^+^ T cells residing in the tongue tumors expressed the immune checkpoint receptor PD-1 compared to those isolated from flank tumors (Fig. 2b). Gene expression analysis by real-time quantitative PCR assay also confirmed relatively higher levels of PD-L1 mRNA in tongue versus flank tumors (Fig. 2c). Furthermore, immunohistochemistry shows significantly higher levels of PD-L1 protein expression in tongue tumors compared to that seen in flank tumors (Fig. 2d, e). Together, these data suggest a close relationship between PD-1/PD-L1 expression level within a given tumor site and responsiveness to α-PD-1 therapy, which is consistent with reported data from human clinical trials in HNSCC as well as several other cancers [17–19].

### Efficacy of α-PD-1 therapy is enhanced by combination treatment targeting CTLA-4

**Fig. 3:**
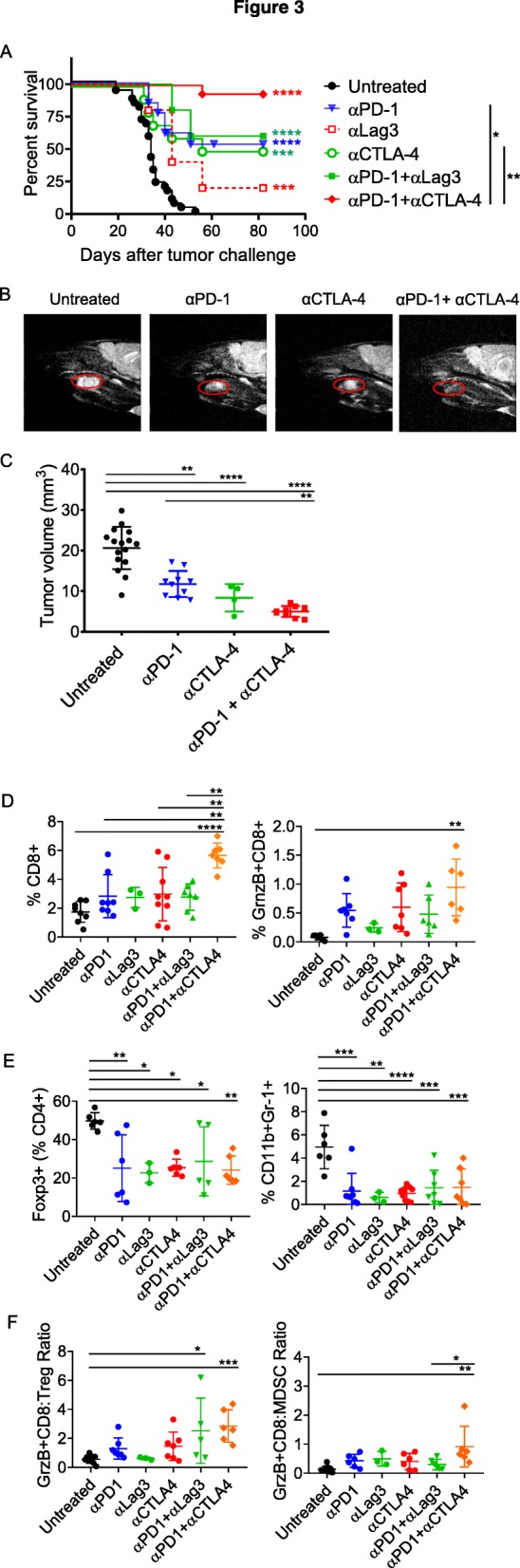
Efficacy of α-PD-1 therapy of tongue-implanted mEER tumors is enhanced by combination treatment with α-CTLA-4 but not α-Lag3. Mice were challenged with mEER tumor cells (4 × 10^4^) in the tongue and treated with antibodies targeting individual checkpoint receptors PD-1, CTLA-4, or Lag3 or using combinations of α-PD-1 and α-CTLA-4 or α-PD-1 and α-Lag-3 antibodies. The percentages of mice surviving in the different groups are shown (**a**). Statistical significance was calculated using Log-rank (Mantel-Cox) test. The significant difference for each treatment group compared to untreated control group is indicated by colored stars and between groups is shown on the legend; *p < 0.05, **p < 0.01, ***p < 0.001, ****p < 0.0001. Tongue tumor volume was measured by MRI (T2-weighted sagittal image) at day 19 after tumor implantation and representative data is shown for one mouse in each group (**b**) along with group means ± SD (*n* = 4–16 mice/group) (**c**). **p < 0.01, ****p < 0.0001, one-way ANOVA. Flow cytometry analyses of TIL isolated at day 15 from tongue tumor-bearing mice subjected to different treatments showing frequencies of total CD8^+^ T cells, Granzyme B expressing CD8^+^ T cells (**d**), CD4^+^FoxP3^+^ Treg, CD11b^+^Gr-1^+^ MDSC (**e**) as well as ratios of GrnzB^+^CD8^+^ T cells to Treg and to MDSC (**f**). Data shown are mean + SD from two experiments (except for anti-Lag3 group) with individual data points representing pooled TILs of 2–3 tumors. Statistical significance was calculated using one-way ANOVA with Turkey post-hoc test; *p < 0.05, **p < 0.01, ***p < 0.001, ****p < 0.0001

In addition to the differential PD-1 expression in tongue- and flank-implanted mEER tumors, CD8^+^ T cells from tongue tumors showed higher levels of additional immune checkpoint inhibitory molecules, CTLA-4 and Lag3 (Additional file 1: Figure S1). We therefore, tested whether combination therapy to block either of these inhibitory receptors would enhance the efficacy of α-PD-1 therapy of tongue tumors. For this, we treated mice with tongue-implanted tumors at days 5, 8 and 11 with α-PD-1 alone or in combination with α-CTLA-4 or α-Lag3. The majority of mice treated with the combination of α-PD-1 and α-CTLA-4 exhibited tumor free survival through day 80 of follow up, while all of the mice in the control untreated group exhibited high tumor burden (Fig. 3a). In contrast, survival rates for mice treated with the combination of α-PD-1 plus α-Lag3 were not significantly different from those of mice treated with α-PD-1 alone. Monotherapy with α-CTLA-4 resulted in a survival advantage similar to that seen with α-PD-1, while targeting Lag3 alone was relatively ineffective (Fig. 3a). The MRI data of head and neck regions of mice collected at day 19 clearly showed significantly reduced tumor size in mice treated with the combination of α-PD-1 and α-CTLA-4, relative to treatment with either antibody alone or untreated mice (Fig. 3b and c), further supporting the positive survival outcome. These data demonstrate that efficacy of α-PD-1 therapy in the tongue implanted mEER tumors can be significantly enhanced by supplementing with immune checkpoint blockade targeting CTLA-4.

To identify the immune cell subsets contributing to the outcome of the combination of α-PD-1 with other immune checkpoint antibodies (α-CTLA-4 or α-Lag3), we analyzed the TIL by flow cytometry at day 15 after tumor implantation (Fig. 3d-f). The frequency of CD8^+^ T cells was significantly increased in the tumors of mice receiving both α-PD1 and α-CTLA-4 compared to no treatment or either antibody alone or the combination of α-PD1 and α-Lag3 (Fig. 3d). Moreover, Granzyme B expression on CD8^+^ T cells was significantly elevated only in the tumors of mice treated with the combination of α-PD1 and α-CTLA-4 relative to control untreated mice (Fig. 3d). Frequencies of immunosuppressive Tregs and MDSC were significantly decreased with all immune checkpoint monotherapies and combinations used, when compared to untreated control (Fig. 3e). Notably, ratios of GranzymeB expressing CD8^+^ T cells (CTL) to Tregs as well as to MDSCs in mice treated with the combination of α-PD1 and α-CTLA-4 were significantly higher when compared to those in control untreated mice (Fig. 3f). Importantly, the most efficacious treatment consisting of the combination α-PD1 and α-CTLA-4 was not toxic in terms of the serum levels of the liver transaminases AST and ALT which fell within the normal range (Additional file 1: Figure S2).

### Intratumoral STING agonist treatment sensitizes multi-focal mEER tumors to checkpoint blockade

Even though α-PD-1 monotherapy was ineffective in treating mice with flank-implanted mEER tumors (Fig. 1a), supplementing α-PD-1 therapy with α-CTLA-4 resulted in regression of 40% of sub-cutaneous mEER and a significant survival advantage (Additional file 1: Figure S3). Since type I and type II interferons (IFNs) are known inducers of PD-L1 expression, which is recognized as a biomarker for α-PD-1 responsiveness on a variety of tumor cells [20, 21], we performed RNA-seq analysis and identified that an IFN pathway signature (both type I and type II) was activated at a significantly higher level in tongue-implanted mEER tumors relative to those on the flank (Additional file 1: Figure S4A and B). This is consistent with the PD-1/PD-L1 expression patterns (Fig. 2) as well as the relatively superior responsiveness of tongue tumors to α-PD-1 therapy (Fig. 1). Based on this information, we reasoned that treatment with type I and/or type II IFNs would improve the α-PD-1 therapy sensitivity of mEER tumors by modulating the expression of PD-1/PDL-1. Additionally, since the cytosolic nucleic acid sensor, Stimulator of Interferon Induced Genes (STING) activates IFN secretion [22], and intratumoral administration of cyclic dinucleotide (CDN) STING agonists such as ML-RR-CDA (ADU-S100) has been shown to activate both IFN-α/β and IFN-γ signaling pathways [23, 24], we tested whether stimulation of the STING pathway would induce expression of PD-1/PD-L1 to promote responsiveness to α-PD-1 therapy. We first performed in vitro stimulation of mEER tumor cells with IFN-α, IFN-γ or ML-RR-CDA, and observed increased PD-L1 expression in response to these treatments (Additional file 1: Figure S4C and D). We and others have shown previously that intratumoral delivery of STING agonist is effective in inducing local as well as systemic anti-tumor immune responses [25, 26]. Therefore, we investigated intratumoral STING agonist treatment as a strategy to reverse the non-responsiveness of flank-implanted mEER tumors to α-PD-1 therapy concurrent with maintaining or improving the antitumor efficacy of α-PD-1 therapy in tongue tumors.

**Fig. 4:**
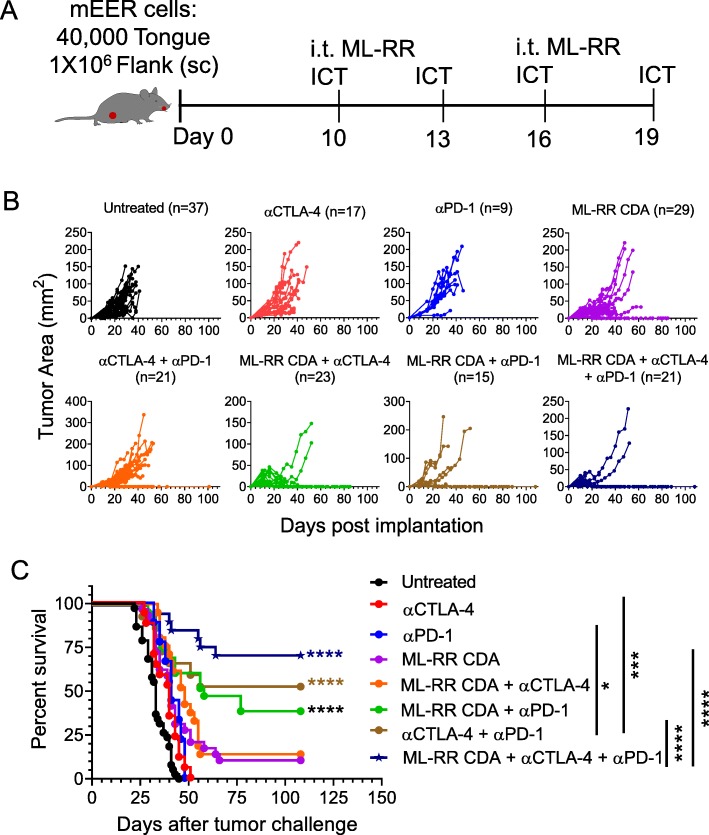
Abscopal anti-tumor efficacy of intratumoral STING activation in combination with systemic checkpoint antibodies. Mice were inoculated with mEER tumor cells both in the flank (1 × 10^6^) and tongue (4 × 10^4^) and treated with intratumoral (i.t.) administration of STING agonist (ML-RR CDA) on days 10 and 16 along with or without immunotherapy employing individual or combinations of α-PD-1 and α-CTLA-4 antibodies on days 10, 13, 16, and 19 (**a**). Growth of flank-implanted tumors over time for individual mice in different treatment groups is expressed in terms of tumor area (mm^2^) in (**b**). The data is pooled from three separate experiments and the total number of mice in each group is noted. The survival curves for mice in different treatment groups are shown in (**c**). Statistical significance for differences in survival of mice in different combination treatment groups relative to untreated control group were calculated using Log-rank (Mantel-Cox) test; *p < 0.05, **p < 0.01, ***p < 0.001, ****p < 0.0001

For these studies, we adopted a pseudometastasic model where mice were implanted with mEER tumors in the tongue as well as on the flank. Different groups of mice were treated with injection of STING agonist into the flank tumors as a monotherapy or in combination with systemic α-PD-1 and/or α-CTLA-4 treatment (Fig. 4a). We observed that intratumoral injection of STING agonist induced complete regression of flank tumors when combined with α-PD-1, or α-CTLA-4 or both together in the majority of mice (Fig. 4b). Importantly, the majority of the mice receiving the combination of intratumoral STING agonist and both systemic α-PD-1 and α-CTLA-4 therapy exhibited a significant survival advantage and clearance of both the flank (Fig. 4c) and tongue tumors (Additional file 1: Figure S5).

**Fig. 5:**
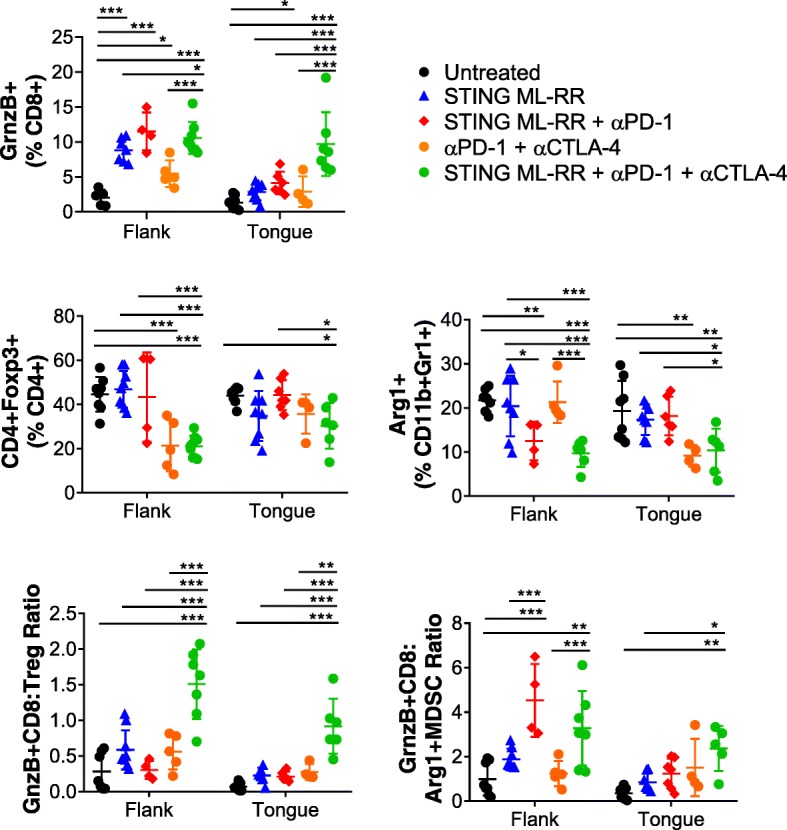
Analysis of immune correlates for combination immunotherapy in the mEER flank-tongue pseudometastasic model. Mice were treated as in Fig. 4a except for ICT antibody administrations performed only on days 10, 13 and 16. Leukocytes isolated from both flank and tongue tumors at day 18 after tumor implantation were analyzed by flow cytometry. Frequencies of total CD8^+^ T cells, Granzyme B^+^ CD8^+^ T cells (CTL), Treg, Arg1^+^ MDSC as well as ratios of CTL to Treg and Arg1^+^ MDSC are shown. Results represent pooled data from two experiments (*n* = 8–14). Statistical significance was calculated using two-way ANOVA and post-hoc correction performed with controlling for false discovery rate (FDR); * < 0.05, ** < 0.01, *** < 0.001

We assessed the immune correlates associated with the observed abscopal therapeutic efficacy of targeting the STING pathway in combination with checkpoint modulation in this pseudometastasic model by performing TIL analysis on day 18 post-tumor implantation (Fig. 5). For TIL analysis, mice were treated as in Fig. 4a except for a total of three ICT treatments on days 10, 13 and 16. We observed that administration of the STING agonist ML-RR-CDA into flank tumors as a monotherapy resulted in a significant increase in the frequency of CTL (Granzyme B expressing functional CD8^+^ T cells) only in flank tumors, but that combined STING agonists and systemic α-PD-1 antibody treatment increased intratumoral CTL in both the flank and the tongue tumors relative to untreated mice. Similarly, while the combination of checkpoint antibodies was able to significantly enhance CTL levels in the flank, CTL frequency in the tongue was only enhanced in combination with STING injection into the flank tumor. Furthermore, we observed that supplementing α-PD-1 + α-CTLA-4 treatment with STING agonist administration into the flank tumors was associated with a decrease in the frequencies of CD4^+^Foxp3^+^ Treg as well as of MDSC expressing Arginase 1 in both flank and tongue tumors. Consequently, the ratios of CTLs to Tregs and to Arg1^+^ MDSC were significantly enhanced with the triple combination therapy of ML-RR-CDA administration into the flank tumor combined with systemic α-PD-1 and α-CTLA-4. These results suggest that intratumoral STING agonist therapy augments the capacity of systemic checkpoint blockade to mediate both tumor regression and survival in a multi-focal model of HPV^+^ HNSCC. Furthermore, pro-inflammatory modulation of the tumor microenvironment of both the STING agonist-injected and uninjected lesions is evident in this model in the context of checkpoint blockade.

## Discussion

We present here, the results from an established HPV^+^ mouse model of oral cancer which illustrates the differential response to immune checkpoint therapy based on the anatomical location of the tumor. While α-PD-1 treatment was effective against orthotopic (tongue) tumors, the same therapy failed to impact those implanted subcutaneously on the flank. These data parallel those reported in a murine lung cancer model (CMT167), where targeting PD-1/PD-L1 interaction caused regression in orthotopic lung tumors by 95%, but only by 30% in flank tumors [27]. Importantly, clinical studies reported higher response rates in primary oral and oropharynx tumors to checkpoint blockade relative to those in distant metastatic disease [28–33].

Even though tongue implanted mEER tumors were more responsive to α-PD-1 therapy compared to poorly responsive flank tumors, overall survival remained only about 50% (Fig. 1a). Based on the high levels of CTLA-4 and Lag3 expressed on CD8 T cells in these tumors, we pursued combination immunotherapy targeting these two additional inhibitory receptors to further improve the effectiveness of α-PD-1 therapy against orthotopic HNSCC. Our results showed that combining α-PD-1 with α-CTLA-4 antibody, but not α-Lag3, resulted in significantly enhanced tumor-free survival relative to that of mice treated with either antibody alone. While the combination of α-PD-1 with α-Lag3 was highly efficacious in preclinical models of fibrosarcoma, colon cancer, and advanced recurring melanoma, the survival advantage was only modest in other cancers such as ovarian cancer [34, 35]. The α-PD-1 and α-CTLA-4 checkpoint blockade therapies are functionally non-redundant and complementary with distinct underlying cellular mechanisms [36]. Moreover, α-CTLA-4 acts both to augment the effector T cells and, simultaneously to numerically and functionally compromise the Treg compartment thus promoting anti-tumor immunity [37]. In contrast, the primary target of α-Lag3 antibodies appears to be a subset of exhausted or inactive CD8 T cells co-expressing Lag3 and PD-1 [35, 38]. Moreover, we observed that mEER tongue tumors were relatively resistant to α-Lag3 monotherapy as compared to α-PD-1 or α-CTLA-4. It is possible that unlike α-PD-1 or α-CTLA-4 antibodies, Lag-3 blockade is more relevant in the early priming phase of T cell activation [39]. Alternatively, although Lag-3 is present on these tumor T cells, its actual capacity to repress their activation and function may be limited in comparison to that of CTLA-4 and PD-1.

Even though, immunotherapy with checkpoint antibodies produces remarkable and durable anti-tumor immune responses, they are associated with acute toxicities in some patients, including a variety of hepatic pathologies attributable to infiltration of immune cells into liver [40, 41]. In the current study, we observed that the levels of liver transaminases in the sera of mice subjected to α-PD-1 treatment in combination with α-Lag3 or α-CTLA-4 remained within the normal range (Additional file 1: Figure S2). It should be noted that the rate of grade 3 or higher AST and ALT elevation was reported to be only 6–9% in Ipilimumab/Nivolumab combination human trial [42]. These low rates of liver toxicity may be harder to see in mice due to lack of external immune exposure history. However, with 4-1BB agonist antibodies like Urelumab, where the liver toxicity rates are much higher in humans, we could see that reflected in mEER tumor model as reported in our previous study [10].

In mEER tongue tumors sensitive to α-PD-1 therapy, we observed an enhanced IFN gene expression signature (both type I and type II), and higher PD-1 levels on CD8^+^ TIL compared to the resistant flank tumors. This profile of enhanced IFN signature and PD-1/PD-L1 expression is consistent with biomarkers identified in KEYNOTE trials and clinical studies of HPV^+^ head and neck cancer [17, 30–33, 43, 44]. Even though, IFN-α therapy is FDA approved for several hematologic and solid tumors, its success and wider application has been restricted due to a complex and non-specific activity profile and significant toxicity. However, activation of STING signaling has emerged as a novel and effective strategy for targeting IFN pathways to positively regulate anti-tumor immune responses [23, 45, 46]. A recent report from our group evaluating intratumoral delivery of checkpoint antibodies targeting CTLA-4, PD-1, and 4-1BB in combination with low-dose STING agonist in the TRAMP-C2 model of prostate cancer showed abscopal tumor regression with combination efficacy correlating with systemic antitumor immune responses [26]. In the current study, we sought to simultaneously target the IFN pathway using a STING agonist along with additional checkpoint modulation (α-CTLA-4) to overcome resistance to α-PD-1 treatment in the flank implanted mEER tumors, while seeking to also enhance the observed efficacy in mice co-implanted with tongue tumors. Intratumoral STING activation along with a combination of α-CTLA-4 and α-PD-1, relative to no treatment or individual treatments, produced the most significant survival advantage in this pseudometastasic setting with regression of both flank and distant tongue tumors. We have shown that such unprecedented abscopal efficacy was associated with a marked increase in the ratios of CTL to Treg as well as to functional MDSC populations. Our results are consistent with a previous report in another oral cancer model where STING agonist was found to be effective against immunogenic, T cell-inflamed MOC1 tumors, and its combination with anti-PD-L1 was able to produce systemic anti-tumor immune responses and regression of bilateral flank tumors [47]. However, STING agonist was ineffective against the related but poorly immunogenic MOC2 tumors [47]. In the pseudometastasic model where we tested the efficacy of intratumoral STING activation along with systemic therapy with the combination of α-PD-1 and α-CTLA-4, we did not observe liver toxicities with single agents or combinations in terms of serum levels of liver transaminases (Additional file 1: Figure S6). The therapeutically effective dose of α-PD-1/α-CTLA-4 and ML-RR-CDA used in our study translate to human equivalent doses comparable to those currently being used in patients [3, 48]. Combining STING agonist with α-PD-1 was almost as effective as α-PD-1 and α-CTLA-4 combination. This is particularly interesting, and the combination of targeting STING along with α-PD-1 could be a better alternative in humans because of relatively higher toxicities expected with α-PD-1 and α-CTLA-4 combination. On the flip side, although intratumoral delivery of STING has been successfully carried out in human trials with accessible solid tumors, it could be challenging depending on cancer type and location. To overcome this, non-nucleotide STING agonists have been developed recently which can be administered systemically [49].

Admittedly, as with many preclinical mouse models, the mEER tongue tumor model showing 50% efficacy of α-PD-1 therapy does not truly mirror the less than 20% human clinical responses. On the other hand, however, PD-1 antibody response rates of mEER tumors in the flank are 0%, far less than the human response rates. Therefore, our primary focus was to understand the underlying mechanisms governing response versus resistance in each site, as a way to gain insight into tissue factors which may dictate differential responses between responder and non-responder patients, and, in turn, to study interventions (i.e. STING agonist) that can push non-responders over into responders. Our results support the use of mEER as a model to test mechanisms involved in α-PD-1 resistance and to identify immunotherapies or their combination with other targeted therapies to enhance the efficacy of α-PD-1 treatment in oropharyngeal tumors.

In conclusion, our results suggest that the therapeutic efficacy of systemic α-PD-1 immunotherapy of HPV^+^ oropharyngeal HNSCC, both in the case of primary and advanced metastatic disease (modeled here with mice harboring tumors in the flank and tongue) can be greatly enhanced by combining with additional T cell checkpoint-targeting antibodies such as α-CTLA-4 and/or through intratumoral delivery of STING activating agents to achieve near complete and durable tumor regression.

## Supplementary information

**Additional file 1: Figure S1**. Differential expression of additional immune checkpoint inhibitory molecules on CD8^+^ T cells from flank or tongue implanted mEER tumors. **Figure S2**. Quantitative analyses of liver enzymes as a function of toxicity of immunotherapy. **Figure S3**. Efficacy of combination treatment with α-PD-1 and α-CTLA-4 against flank- implanted mEER tumors. **Figure S4**. Elevated Type I and Type II IFN signaling and PD-L1 expression in tongue-implanted mEER tumors relative to those on the flank. **Figure S5**. Efficacy of combination immunotherapy on the growth of tongue-implanted mEER tumors. **Figure S6**. Immunotherapy with STING agonist (ML-RR-CDA) alone or in combination with different checkpoint antibodies in mEER pseudometastasic model is not toxic.

## Data Availability

Data and material presented in this study are available upon request.

## Abbreviations

ALT: Alanine transaminase
ANOVA: Analysis of variance
Arg1: Arginase 1
AST: Aspartate transaminase
CDA: Cyclic di-adenosine
CDN: Cyclic dinucleotide
CTL: Cytolytic T cell
CTLA-4: Cytotoxic T-lymphocyte-associated protein 4
HED: Human equivalent dose
HNSCC: Head and neck squamous cell carcinoma
HPV: Human papilloma virus
i.p.: intraperitoneal
i.t.: intratumoral
ICT: Immune checkpoint therapy
IFN I: Type I interferon
IFN II: Type II interferon
Lag3: Lymphocyte activating 3
MDSC: Myeloid-derived suppressor cells
MRI: Magnetic resonance imaging
OPC: Oropharyngeal cancer
PD-1: Programmed cell death protein 1
PD-L1: Programmed death-ligand 1
STING: Stimulator of interferon genes
TIL: Tumor infiltrating lymphocytes
