# Supplementary material for: Targeting interferon signaling and CTLA-4 enhance the therapeutic efficacy of anti-PD-1 immunotherapy in preclinical model of HPV+ oral cancer
Source: J Immunother Cancer. 2019 Sep 18;7:252. doi: 10.1186/s40425-019-0728-4 (PMC6749627; doi:10.1186/s40425-019-0728-4)
Supplement: Supplementary file 1 — Additional file 1: Figure S1. Differential expression of additional immune checkpoint inhibitory molecules on CD8+ T cells from flank or tongue implanted mEER tumors. Figure S2. Quantitative analyses of liver enzymes as a function of toxicity of immunotherapy. Figure S3. Efficacy of combination treatment with α-PD-1 and α-CTLA-4 against flank- implanted mEER tumors. Figure S4. Elevated Type I and Type II IFN signaling and PD-L1 expression in tongue-implanted mEER tumors relative to those on the flank. Figure S5. Efficacy of combination immunotherapy on the growth of tongue-implanted mEER tumors. Figure S6. Immunotherapy with STING agonist (ML-RR-CDA) alone or in combination with different checkpoint antibodies in mEER pseudometastasic model is not toxic. [file 40425_2019_728_MOESM1_ESM.pdf]

Supplemental Figures

Figure S1

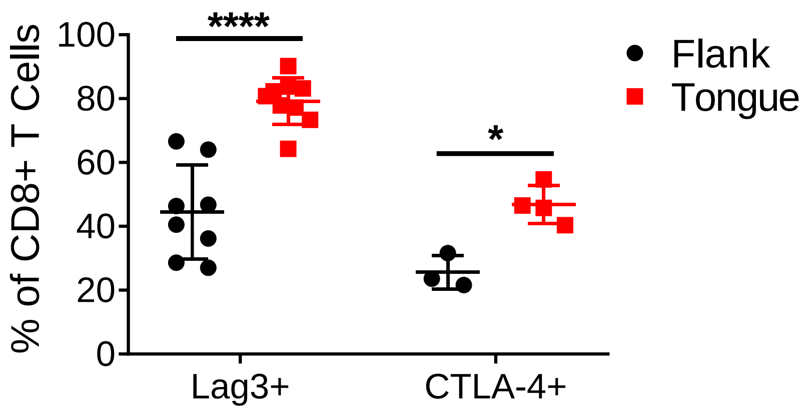

Figure S1: Differential expression of additional immune checkpoint inhibitory molecules on $CD8^+$ T cells from flank or tongue implanted mEER tumors. Tumor-infiltrating leukocytes isolated on day 19 after implantation of tumors in the flank or tongue were analyzed by flow cytometry for checkpoint molecule expression, and percentages of $CD8^+$ T cells expressing Lag3 or CTLA-4 are shown. Individual data points are shown along with group mean $\pm$ SD. Results represent pooled data from two experiments (n = 6-18). Statistical significance was calculated using two-way ANOVA with Sidak post-hoc correction; *$p < 0.03$, ****$p < 0.0001$.

Figure S2

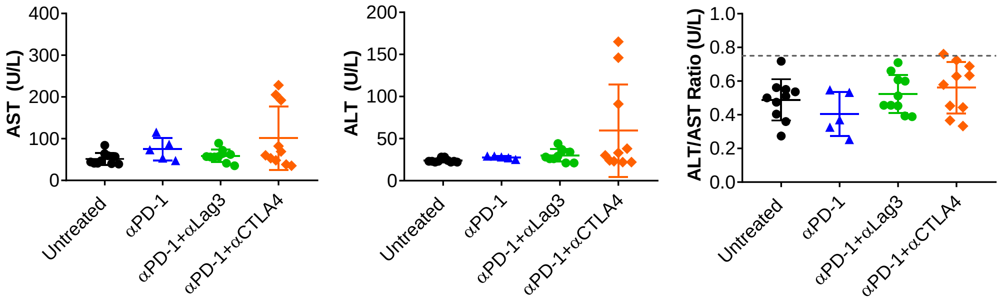

Figure S2: Quantitative analyses of liver enzymes as a function of toxicity of immunotherapy. Serum samples from in different treatment groups were collected on day 19 and analyzed for the levels of AST and ALT as well as the ratio of AST to ALT. Data points represent values for individual mice from each treatment group (n=5-10). Dashed line highlights the upper limit of normal range.

Figure S3

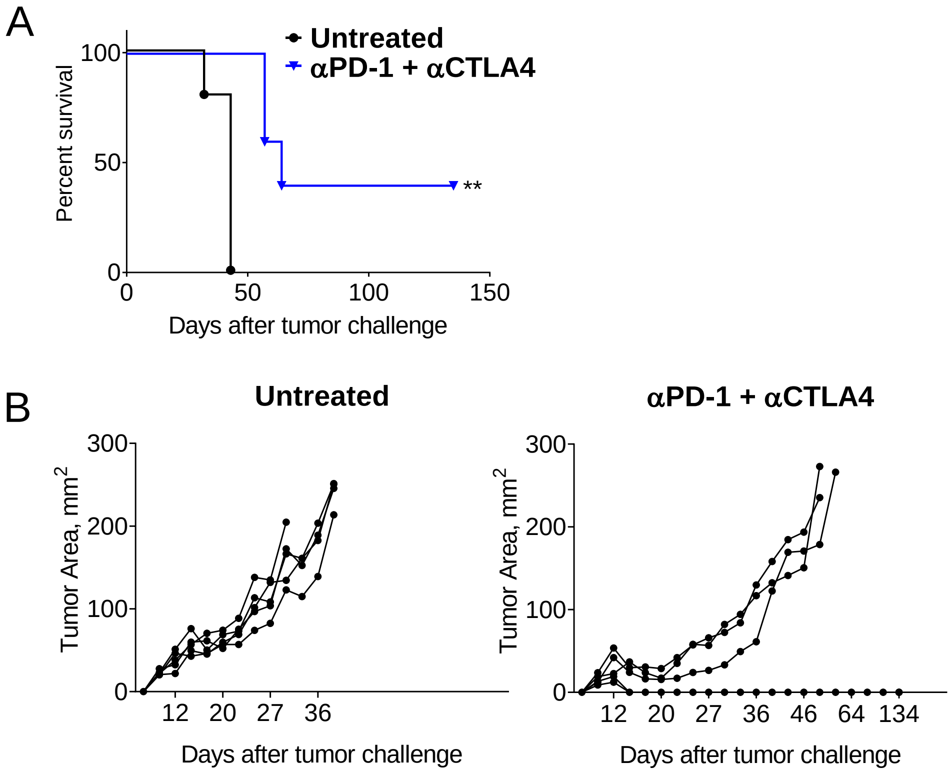

Figure S3: Efficacy of combination treatment with α-PD-1 and α-CTLA-4 against flank-implanted mEER tumors. Mice were injected with tumor cells (1x106) subcutaneously in flanks and were either untreated or treated with the combination of α-PD-1 and α-CTLA-4 antibodies on days 5, 8 and 11 (n=5). Survival of mice in the two different groups (A) and the tumor size in terms of tumor area (mm2) for individual mice in each group (B) were monitored twice weekly. Statistical significance was calculated using Log-rank (Mantel-Cox) test, **p < 0.01.

Figure S4

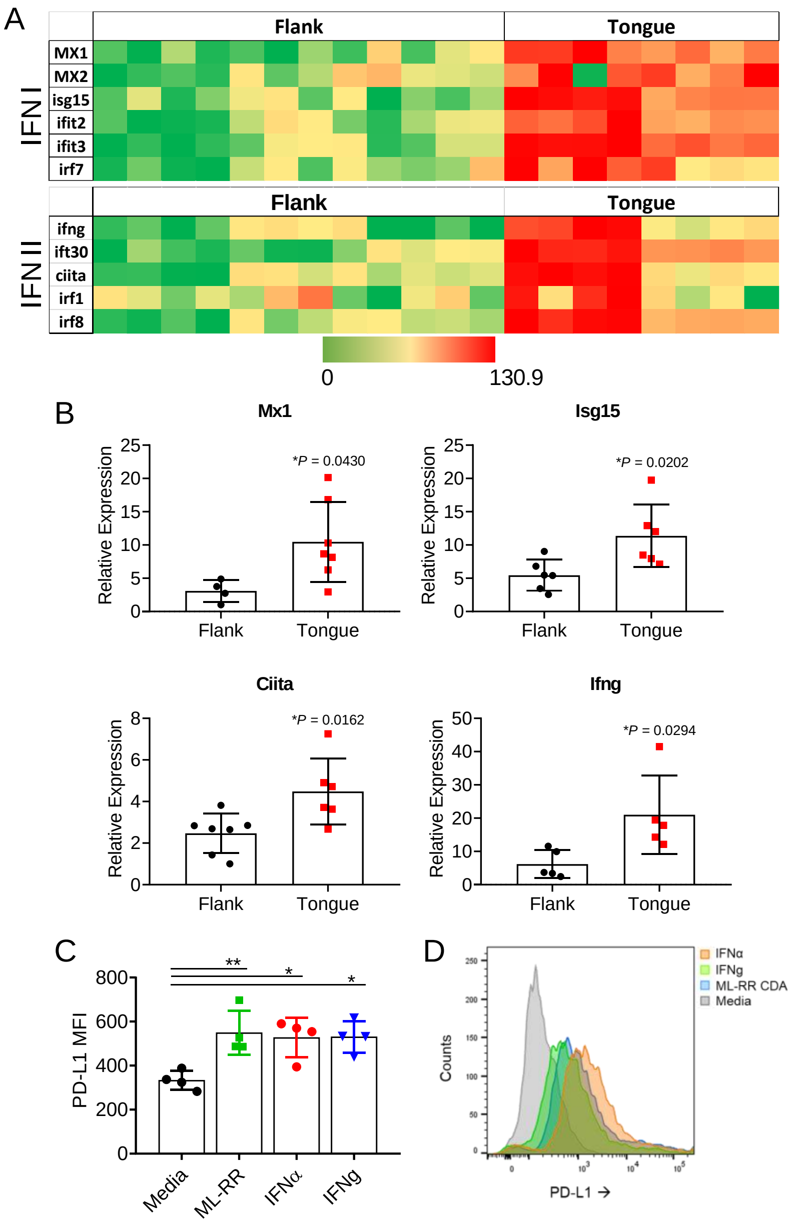

Figure S4: Elevated Type I and Type II IFN signaling and PD-L1 expression in tongue-implanted mEER tumors relative to those on the flank. The heat maps show RNASeq analyses of flank and tongue implanted mEER tumors (A) for the expression IFN I- and II-associated genes. Validation for the differences in the expression levels of selected genes was performed using real-time qPCR analysis (B). Data represent mean $\pm$ SD from 4-7 mice per group. Statistical significance was calculated using Student's *t*-test and *P* values are shown. C) The mEER tumor cells treated *in vitro* with IFN-α (25 ng/mL), IFN-γ (25 ng/mL) or STING agonist ML-RR-CDA (0.5 ug/mL) exhibit elevated PD-L1 expression as analyzed by flow cytometry and the mean fluorescence intensity (MFI) values $\pm$ SD for 4 replicate treatments (C) along with representative histograms for PD-L1 expression (D) are shown. *$p < 0.05$, **$p < 0.005$ based on one-way ANOVA with Dunnet post-hoc correction.

Figure S5

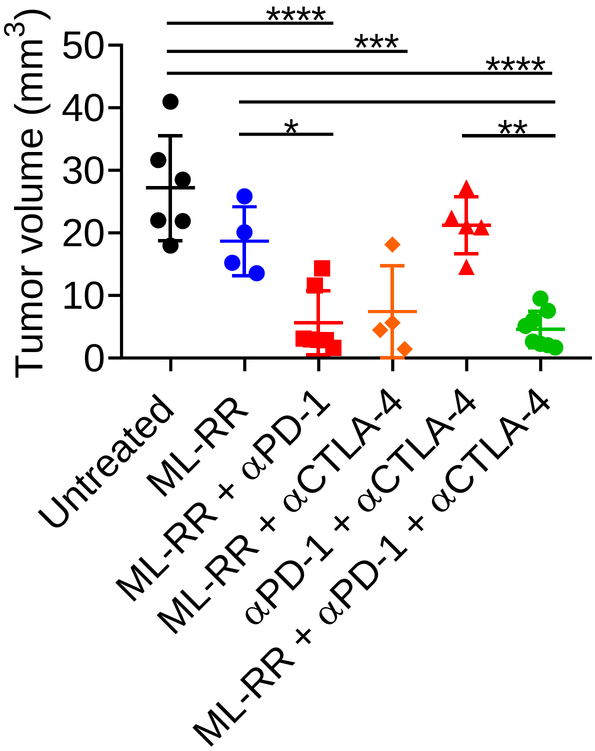

Figure S5: Efficacy of combination immunotherapy on the growth of tongue-implanted mEER tumors. Mice were implanted with mEER tumors both in the flank and the tongue, and different treatments performed as described in Figure 4. The head and neck regions of individual mice were subjected to MRI on day 23 and tumor volumes were calculated as described in Methods. Data shown are values for individual mice with group means ± SD (n = 4-8 mice/group). *$p < 0.05$, **$p < 0.01$, ***$p < 0.001$, ****$p < 0.0001$ based on one-way ANOVA with Tukey post-hoc correction.

Figure S6

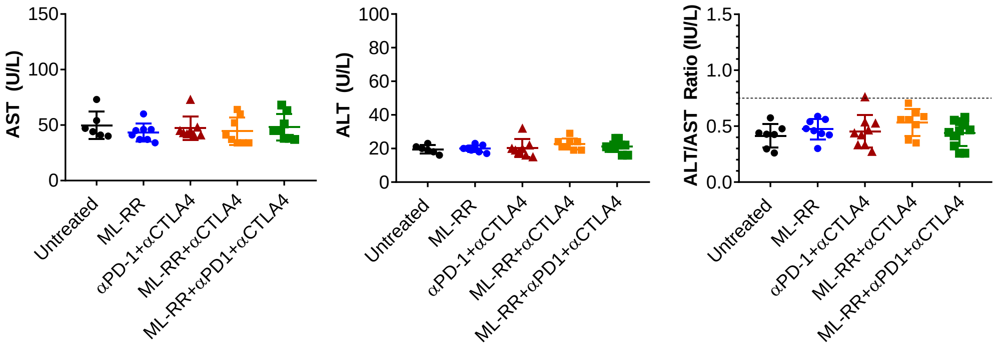

Figure S6: Immunotherapy with STING agonist (ML-RR-CDA) alone or in combination with different checkpoint antibodies in mEER pseudometastasic model is not toxic. Mice were implanted with mEER tumors both in the flank and the tongue, and treated as shown in Figure 4. Serum samples collected on day 21 were analyzed for the levels of AST and ALT as well as the ratio of ALT to AST as shown. Data points represent values for individual mice from each treatment group shown with mean ± SD (n=6-10 from two experiments). Dashed line marks the upper limit of normal range.
